# A longitudinal path analysis of the reciprocal and cyclical relationships between sickness absence, job demands, job resources, and burnout

**DOI:** 10.3389/fpsyg.2025.1557898

**Published:** 2025-04-04

**Authors:** Simon Gottenborg, Thomas Hoff, Svein Åge Kjøs Johnsen, Leif Rydstedt, Kjell Ivar Øvergård

**Affiliations:** ^1^Department of Psychology, Inland School of Business and Social Sciences, University of Inland Norway, Lillehammer, Norway; ^2^EBHR AS, Oslo, Norway; ^3^Department of Psychology, University of Oslo, Oslo, Norway; ^4^Department of Health, Social, and Welfare Studies, Faculty of Health and Social Sciences, University of South-Eastern Norway, Borre, Norway

**Keywords:** sickness absenteeism, job demands, job resources, exhaustion, longitudinal, path analysis

## Abstract

**Aim:**

This study investigates the longitudinal reciprocal cyclical impact of sickness absence on perceived job demands and job resources, as well as its indirect effects on future burnout and further sickness absence.

**Design and methods:**

A four-wave longitudinal survey design was employed, with sickness absence data collected at Time 1 and Time 3 and questionnaires assessing psychosocial work environment factors administered at Time 2 and Time 4.

**Sample:**

A total of 272 employees from several Norwegian organizations participated in the study.

**Results:**

The results provided evidence of a reciprocal longitudinal negative path coefficient between sickness and perceived job resources, while the path coefficient related to job demands was small and non-significant. Additionally, a cyclical reciprocal effect was identified, following the pathway: sickness absence -> job resources -> burnout -> sickness absence, thereby supporting the JD-R model’s predictive capability regarding sickness absenteeism. This implies that sickness absence may lead to a perceived loss of job resources, which subsequently exacerbates burnout and results in further sickness absence over time.

**Contribution:**

This study contributes to psychological theory by enhancing the understanding of the longitudinal and reciprocal effects of sickness absence on perceived job characteristics. It also expands the longitudinal evidence base demonstrating burnout’s predictive effect on sickness absence.

## 1 Introduction

Sickness absenteeism refers to the loss of work hours due to employee illness or injury. At the societal level, sickness absenteeism is a costly affair. In the EU in 2019, an average of 12.6 days per employee per year were lost due to illness or injury (WHO, 2022). Sickness absence differs between countries; in the United States in 2021, sickness absenteeism led to an average work absence rate of 3.2%, while Norway recorded an average of 6.2%, or approximately 14.3 workdays per employee per year in the same year (SSB, 2022).

Sickness absence leads to increased organizational costs and decreased productivity. In a 2010 survey of Norwegian organizations, managers estimated the average cost of 1 week of sickness absence per worker (Hem, 2011) at NOK 13,000. After adjusting for inflation, this would translate to a 2022 cost of NOK 17109 (ca. €1,630) per week per employee.

Sickness absenteeism is also associated with reduced production (Ybema et al., 2016). A recent study estimated that a 1% increase in sickness absenteeism was associated with an average production loss of 0.66%. This effect is more pronounced in blue-collar occupations compared to white-collar occupations, particularly when long-tenured workers are sick (Grinza and Rycx, 2020).

On an individual level, research has shown several negative effects of sickness absence. First, the duration of sickness absence is negatively associated with salary and career development (Judiesch and Lyness, 1999; Sieurin et al., 2009), as well as with an increased probability of future unemployment (Hesselius, 2007). Second, sickness absence negatively affects employees’ workability by increasing perceived physical and mental job demands (Gustafsson and Marklund, 2011). Third, past sickness absence predicts future sickness absence (Laaksonen et al., 2013; Reis et al., 2011). Finally, sickness absenteeism may also reduce access to salutogenic aspects of work. Those suffering from long-term sickness absenteeism have a higher risk of becoming socially and economically disadvantaged (Bryngelson, 2009).

Work can also have positive effects, which are lost when one cannot work due to illness. Several theoretical and empirical lines follow the idea that work has salutogenic factors, where work is seen as contributing to authenticity, self-efficacy, self-esteem, purpose, belongingness, transcendence, and sensemaking, all of which contribute to individual well-being (Rosso et al., 2010). In another line of reasoning, there is Jahoda’s (1982) theory of mental health, where work provides several factors necessary for well-being and mental health, such as (1) *time structure*, where work helps structure an employee’s life and fill the day with planned goal-oriented activities. When people cannot go to work, this structure is broken “*days stretch long when there is nothing that has to be done; boredom and waste of time become the rule*” (Jahoda, 1982), p. 28; (2) work also contributes with *collective purpose* where working together toward a common goal creates a sense of usefulness and purpose that brings meaning to the person’s life; (3) *social status*; having a job and doing it well is respected and valued in our society and thus being employed becomes an important part of one’s own identity; and (4) *social relations* outside the immediate family, which is seen to be important as it increases the possibility for meaningful and interesting exchange of information and opinions thus increasing the depth and scope of people’s lives (Jahoda, 1982). Although Jahoda’s theory was originally developed for unemployment, it may also be used as a general theory for the mental health benefits of employment (Paul and Batinic, 2010). Therefore, being away from work for an extended period (whether it is because of illness, unemployment, or other unintended reasons) may deprive employees of these positive aspects of work (Paul and Batinic, 2010).

Thus, sickness absence is not merely about “not having to work” but also about being cut off from a range of important, meaningful, and salutogenic aspects of an adult employee’s life.

### 1.1 The job demands-resources model

The JD-R model is a theoretical framework that has gained much empirical support in the last two decades (Bakker and Demerouti, 2007, 2017). The model proposes that job characteristics and work environment variables can be categorized into two overarching categories named *job demands* and *job resources*, which lead to two psychological processes: one pathological process, which leads to an impairment of health, and a salutogenic or motivational process, which leads to growth and development (Bakker et al., 2014; Jenny et al., 2017).

Job resources are defined as *“… those physical, psychological, social, or organizational aspects of the job that may do any of the following: (a) be functional in achieving work goals, (b) reduce job demands at the associated physiological and psychological costs*; *(c) stimulate personal growth and development.*” (Demerouti et al., 2001), p. 499. Examples of job resources include, for example, factors associated with personal growth (e.g., investment in employee development), constructive feedback on performance, social support, and job autonomy (Gottenborg et al., 2022). The presence of job resources leads to a motivational or salutogenic process that promotes health, personal growth, and well-being (Bakker et al., 2014; Jenny et al., 2017; Langseth-Eide and Vittersø, 2021). Job resources help mitigate the impact of job demands on negative subjective states such as emotional burnout (Bakker et al., 2005; Schaufeli and Taris, 2014).

Job demands are defined as “… *those physical, social, or organizational aspects of the job that require sustained physical or mental effort and are therefore associated with certain physiological and psychological costs*…” (Demerouti et al., 2001), p. 499. Job demands are associated with a psychological strain process leading to impairment of health (Bakker et al., 2014). Job demands are positively correlated with negative subjective states such as burnout (Xanthopoulou et al., 2007). The impact of job demands on burnout is stronger when employees have fewer available job resources (Bakker et al., 2005; Hansen et al., 2009; Xanthopoulou et al., 2007).

The longitudinal associations between job demands, job resources, and burnout have been empirically supported several times (Brough et al., 2013; Lesener et al., 2019). A meta-analysis of longitudinal studies testing the JD-R model found support for the main theoretical predictions of the JD-R model. For high-quality studies, they found a positive path coefficient between job resources and work engagement (*ρ* = 0.18) and between job demands and burnout (*ρ* = 0.13), as well as a negative correlation between job resources and burnout (*ρ* = −0.16), thus supporting the main theoretical claims made by the JD-R model. Interestingly, they also found that models that allowed for reciprocal relationships between job characteristics and well-being had a better fit to the data and, hence, were better able to capture the empirical associations between the observed constructs. Thus, they pointed to the need for more studies investigating reversed or reciprocal relationships, thus focusing on the dynamic nature of the JD-R model (Lesener et al., 2019).

### 1.2 Aim and contributions of this study

Following Lesener et al.’s (2019) call to arms for investigating reversed or reciprocal relationships between variables within the JD-R model framework, we aim to investigate the reversed longitudinal and cyclical relationship between sickness absenteeism, job demands, job resources, and burnout. Our main aim will be to test (1) whether sickness absence has a longitudinal predictive effect on job demands and on job resources and (2) to utilize the well-known and empirically supported relationship between job demands, job resources, and burnout (Lesener et al., 2019) to test a cyclical model where sickness absenteeism at time 1 has an indirect effect on sickness absenteeism at time 2 following these two paths:

(1) Sickness absence T1 → job resources T2 → burnout T2 → sickness absence T3
(2) Sickness absence T1 → job demands T2 → burnout T2 → sickness absence T3

The contribution of this stated aim is clear when we compare this study with other longitudinal research on the relationship between job characteristics, burnout, and sickness absenteeism (Clausen et al., 2012; de Jonge et al., 2010; Kottwitz et al., 2018; Schaufeli et al., 2009; Suominen et al., 2007). All these studies (except for Gustafsson and Marklund, 2011) have focused on the predictive effect of job resources, job demands, or burnout on sickness absenteeism, while only Gustafsson and Marklund have assessed the reciprocal relationships *from* sickness absence *to* other outcomes such as workability, health, and future sickness absenteeism. However, they did not test these relationships within the framework of the JD-R model (Gustafsson and Marklund, 2011).

Thus, the contributions of this study will be (a) testing the JD-R model in a longitudinal design with more data from three more time points [as requested by Lesener et al. (2019)]; (b) testing cyclical models with reversed relationships between components of the JD-R model (e.g., job demands, job resources and burnout) and outcomes (e.g., sickness absenteeism), and (c) presenting more longitudinal evidence of the ability of the JD-R model to predict sickness absenteeism.

### 1.3 The JD-R model and sickness absence

The JD-R model is a generic empirical framework (Schaufeli and Taris, 2014) that can be used to predict a large set of positive and negative outcomes (Bakker and Demerouti, 2017; Bakker et al., 2014). In addition to predicting the relationship between job demands, job resources, burnout, and work engagement, the JD-R model also predicts positive and negative outcomes such as turnover (Leiter and Maslach, 2009; Willard-Grace et al., 2019), sickness absenteeism (Ahola et al., 2008; Bakker et al., 2004; Borritz et al., 2010; Langseth-Eide and Vittersø, 2021; Peterson et al., 2011; Toppinen-Tanner et al., 2005), or positive outcomes such as self-assessed health (Langseth-Eide and Vittersø, 2021) and productivity (Bakker et al., 2004). Schaufeli et al. (2009) found that job resources and job demands were both related to burnout and that burnout had a positive and predictive path coefficient to future absence duration, i.e., the more burned-out an employee was, the longer the absence spells tended to last. Another study has linked the perceived availability of job resources with lower sickness absence (Väänänen et al., 2003). Our first hypothesis will, therefore, be related to a longitudinal test of the JD-R model’s ability to predict sickness absenteeism by using the Burnout scale from COPSOQ II:

*H1a*: Job resources will have an indirect negative effect on sickness absenteeism through burnout.

*H1b:* Job demands will have an indirect positive effect on sickness absenteeism through burnout.

This will be a test to see if our data is in line with the standard model of JD-R and whether this model predicts negative outcomes such as sickness absenteeism.

### 1.4 Reciprocal effects of sickness absence on job demands and job resources

Now, the following hypotheses relate to the reversed causality between sickness absenteeism and job resources and job demands. Following the finding that sickness absenteeism has a negative covariance with workability, which leads to an increase in the perceived physical and mental job demands, (Gustafsson and Marklund, 2011), we expect that the extent of sickness absenteeism will be positively correlated with perceived job demands after we control for job demands at a previous point in time. Thus, our hypotheses H2a and H2b will be regarding the reciprocal effect between sickness absenteeism and job demands:

*H2a*: There will be a positive path coefficient between sickness absence at T1 and job demands at T2.

*H2b*: There will be a positive path coefficient between sickness absence at T3 and job demands at T4 after we have controlled for job demands measured at T2.

Now, as previously mentioned, sickness absenteeism is not only about “not working” or “being away from work.” It is also about being involuntarily away from a job that contains several characteristics important for mental health, such as *social status*, *social relations*, and a sense of *shared purpose* and being part of a social group (Jahoda, 1982; Paul and Batinic, 2010). These elements could be seen to be defined as job resources, and comparable operationalizations are used in such work environment scales, such as “social support from colleagues,” “recognition of your work,” and “feedback on performance” (Gottenborg et al., 2022). Thus, being away from work involuntarily due to sickness deprives people of some of the things that are important for employees if we are to believe Jahoda (1982). This leads to the following hypotheses regarding the reversed causal effects of sickness absence on job resources:

*H3a:* There will be a negative path coefficient between sickness absence at T1 and job resources at T2.

*H3b:* There will be a negative path coefficient between sickness absence at T3 and job resources at T4 after we have controlled for job resources measured at T2.

In addition to evidence of causal relationships between job demands and burnout and job resources and work engagement (references), there are also reciprocal effects between subjective states of subjective states such as engagement, burnout, and depression and the perception of job characteristics. Burnout has been found to predict future work pressure (Demerouti et al., 2004) and other longitudinal studies have found similar reciprocal effects for other job characteristics and burnout (De Lange et al., 2004; Zapf et al., 1996) or for job stressors, depression, and work-home interference (Steinmetz et al., 2008).

### 1.5 Cyclical path between sickness absence, job characteristics, and burnout

Previous episodes of sick leave, regardless of the types of disorders, have been shown to predict the risk of future sickness absence (Laaksonen et al., 2013; Reis et al., 2011). Hence, we expect that sickness absenteeism is autocorrelated. Combining this finding with the possibility of a reciprocal cyclical relationship between sickness absenteeism → job characteristics → burnout → sickness absenteeism, we, therefore, expect that some of the effect from sickness absence on T1 on future sickness absenteeism on T2 to be mediated along two paths, one mediated through job resources → burnout and another mediated through job demands → burnout. This leads to the following hypotheses regarding two different indirect cyclical reciprocal longitudinal effects of sickness absence:

*H4a:* Sickness absence at T1 will have a negative indirect effect on sickness absenteeism at T3 on T2 mediated through the path of job resources → burnout on T2.

*H4b:* Sickness absence at T1 will have a negative indirect effect on sickness absenteeism at T3 on T2 mediated through the path job demands → burnout on T2.

**Figure 1:**
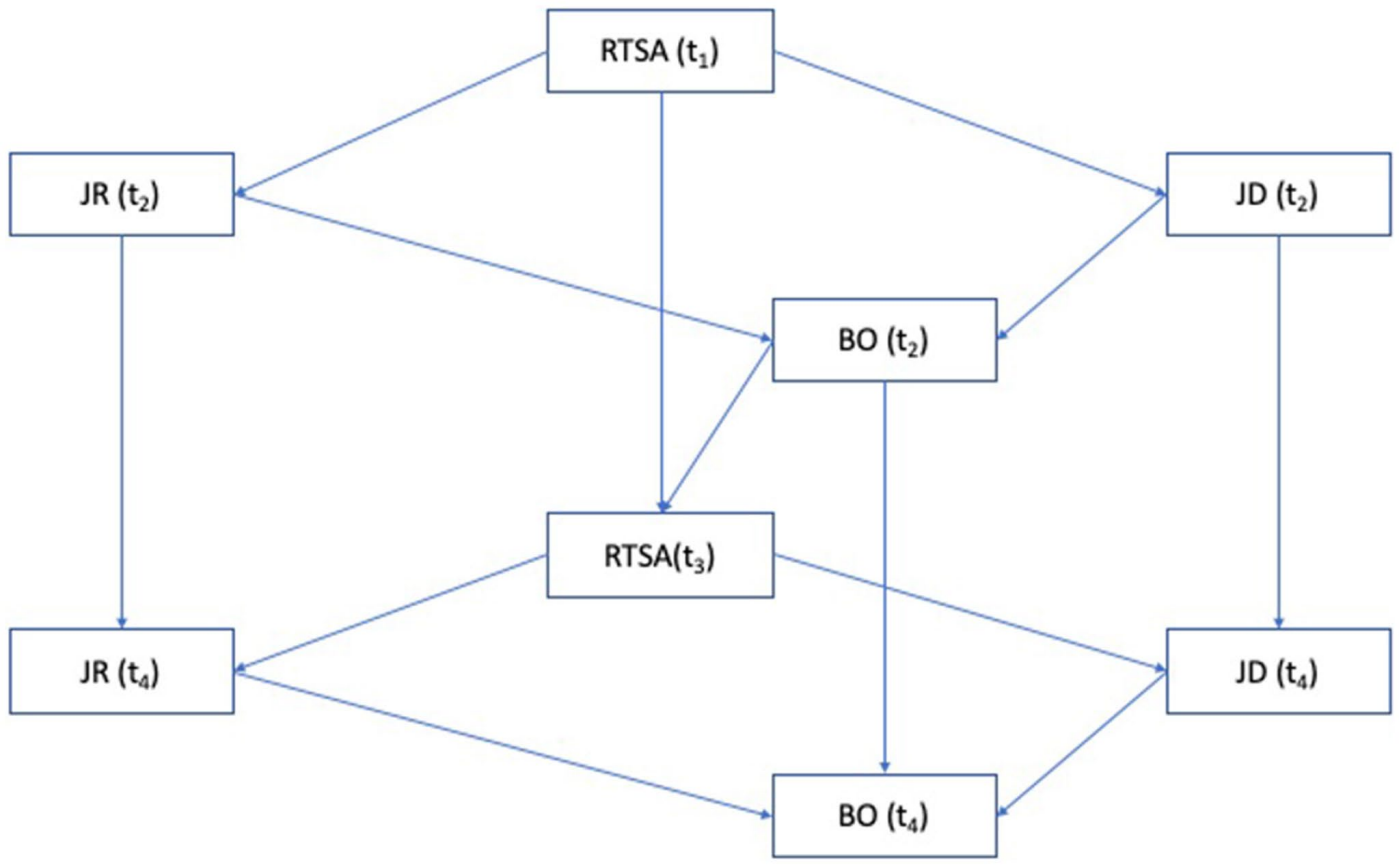
Initial theoretical model. RTSA, Root Transformed Sickness Absence; JR, Job Resources; JD, Job Demands; BO, Burnout.

The initial theoretical path model with all estimated paths is presented in Figure 1.

## 2 Methods

### 2.1 Data collection

This study employs a longitudinal research design with data collected at four separate times (twice for both sickness absence and psychosocial work environment factors). The data used has been collected by EBHR in collaboration with several Norwegian companies. The study includes data on sickness absence between October 2017 and September 2018, as well as between October 2018 and September 2019. Employee surveys were conducted in September 2018 and September 2019. Longitudinal measures can estimate the causal relationships and the predictive validity of a measurement model. This is a significant improvement compared to cross-sectional studies, where one can only measure covariation without being able to attribute any cause-effect effect. The surveys used were largely based on the People-Performance Scales (PPS) questionnaire, which uses 57 items to measure 15 constructs (Gottenborg et al., 2022).

### 2.2 Sample

The original data file had data from 1,010 anonymized respondents. All cases with missing data were removed before proceeding with the analysis, leaving a sample of 272 participants. Most participants were women (70%), and the average age was 42 years (SD = 11.6).

### 2.3 Ethical considerations

Respondents were informed that their answers could be used for research purposes prior to answering the survey in large data sets where anonymity could be guaranteed. All files were made anonymous through the deletion of person-identifiable information prior to use in research, and no code file that can allow backtracking of results to individuals exists. According to Norwegian law, all anonymous data files are free for use for any purpose.

### 2.4 Measures and timeline

**Figure 2:**
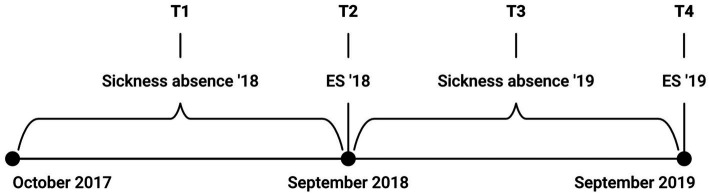
Visual representation of data collection. T1 refers to the measurement of sickness absence from 1 October 2017 to 30 September 2018; T2 refers to the employee survey in September 2018, which measures job resources, job demands, and burnout; T3 refers to the measurement of sickness absence from 1 October 2018 to 30 September 2019; and T4 refers to the employee survey in September 2019. ES, Employee survey.

Four different constructs were of interest: job resources, job demands, burnout, and sickness absence. Data was collected in four different time periods (T1 to T4). The percentage of sickness absence (number of days with sickness absence/number of planned workdays) was calculated for two time periods, T1 between 01.10.2017 and 30.09.2018 and T3 between 01.10.2018 and 30.09.2019. Employee surveys measuring job resources, job demands, and burnout were measured at T2 (September 2018) and T4 (September 2019). A visual representation of the data collection can be seen in Figure 2.

#### 2.4.1 Sickness absence

Sickness absence was measured by the number of workdays lost due to sickness absence. Mean sickness absence on T1 was 9.48 days (SD = 22.42) with a minimum of 0 days and a maximum of 166 days. Skewness and Kurtosis were 4.767 and 24.99, respectively. On T2, the mean sickness absence was 9.80 days (SD = 19.37), with a minimum of 0% and a maximum of 128 days. Skewness and kurtosis were 3.64 and 14.99, respectively. The correlation between Sickness absence at the two measurements was of moderate size (*r* = 0.29, 95% CI [0.18, 0.40]). Skewness was considered too high for values above ±3.0, while kurtosis was considered too high for values above ±10 (Kline, 2011). Skewness and Kurtosis were unacceptable for Sickness absence on both Time 1 (Skewness = 4.59, Kurtosis = 23.1) and Time 3 (Skewness 3.54, Kurtosis = 14.0).

Because of the right-tailed distribution and the excessive skew in combination with the fact that our sickness absenteeism variables contain zeroes, which makes it impossible to use the log-transform $\log\left(x\right)$ or the reciprocal transformation $\left(\frac{1}{x}\right)$, we chose to transform the sickness absenteeism days variables at T1 and T3 with the square root transformation, where each $x_{i}$ is replaced with $\sqrt{x_{i}}$ (Bartlett, 1936). The square root transformation helps reduce skew and linearizes the relationship to other variables (Cohen and Cohen, 1983, p. 236). Both Root Transformed Sickness Absence (RTSA) variables had acceptable skewness and kurtosis (see Table 1) according to the requirements outlined by Kline (2011). Correlations between RTSA T1 and RTSA T2 were of moderate size (*r* = 0.40, 95% CI [0.30, 0.50]). Categorization of the magnitude of effect sizes throughout this article follows Cohen’s classification (Cohen, 1988, 1992).

**Table 1:** Descriptive statistics for variables with correlations.

		M	SD	Sk	Ku	1	2	3	4	5	6	7
1	RTSA T1	2.14	2.29	1.50	2.64	-						
2	RTSA T3	2.08	2.27	2.08	6.10	0.40**	-					
3	Job Demands T2	2.88	0.79	0.05	0.11	0.05	0.04	-				
4	Job Demands T4	2.95	0.83	−0.01	0.06	−0.01	0.08	0.66**	-			
5	Job Resources T2	3.84	0.58	−0.49	0.58	−0.13*	−0.13*	−0.51**	−0.38**	-		
6	Job Resources T4	3.78	0.68	−0.75	1.15	−0.13*	−0.19**	−0.40**	−0.51**	0.67**	-	
7	Burnout T2	2.61	0.99	0.10	−0.81	0.07	0.21**	0.52**	0.44**	−0.47**	−0.39**	-
8	Burnout T4	2.64	1.05	0.14	−0.71	0.06	0.17**	0.44**	0.56**	−0.37**	−0.45**	0.74**

** *p* < 0.01 level (2-tailed), * *p* < 0.05 (2-tailed), cross-correlations of the same variable between two measurement times in bold. *N* = 272 for all variables.M, Mean; SD, Standard Deviation; Sk, Skewness; Ku, Kurtosis; RTSA, Root Transformed Sickness Absence.

#### 2.4.2 Job resources

Due to the small sample size, we used parceling (Little et al., 2002) by aggregating the scores on the following job resource scales: *autonomy*, *feedback*, *leadership quality*, *involvement*, *investment in employee development*, and *support from colleagues*. Job resource scales measured on T2 were summed into job resources T2, and job resource scales measured in T4 were summed into job resources T4. All scales and items are described in a recent publication on the work environment questionnaire People-Performance Scales (PPS) (Gottenborg et al., 2022). Internal consistency was evaluated by calculating Cronbach’s alpha for all constructs at both time periods. All four constructs showed acceptable internal consistency, with alphas ranging from 0.87 (Autonomy at T2) to 0.94 (Feedback at T2). Correlation between job resources at T2 and T4 were large (*N* = 272, *r* = 0.67, 95% CI [0.60, 0.73]).

#### 2.4.3 Job demands

The variable job demands was calculated as parcels (Little et al., 2002) by averaging across the means of the *workload* and *role conflict* scales. The workload scale used was taken from the PPS Questionnaire (Gottenborg et al., 2022), while the role conflict scale consisted of three items based on a scale with the same name from the QPSNordic questionnaire (Elo et al., 2000). Both scales showed acceptable internal consistency, with Cronbach’s alphas ranging from 0.82 (role conflict at time 2 and 4) to 0.87 (workload at time 4). Correlations between job demands at T2 and T4 were large (*N* = 272, *r* = 0.66, 95% CI [0.58, 0.72]).

#### 2.4.4 Burnout

Burnout was measured using the Norwegian translation of the four-item burnout scale from the Copenhagen Psychosocial Questionnaire, Second Edition (COPSOQ II) (Pejtersen et al., 2010). The Norwegian translation is part of the People Performance Scales (PPS), and the scale has good psychometric properties (Gottenborg et al., 2022). The items in this scale are similar in content to items in the Exhaustion facet of the Burnout construct as measured by the Oldenburg Burnout Inventory (Demerouti and Bakker, 2008). We used this measure of Burnout because of the low number of items combined with the good psychometric properties of the scale. The low number of items was necessary because the data collection happened in an applied organizational consultancy format, restricting the number of questions. Hence, the questionnaire needs to be as short as possible while retaining good psychometric properties (Gottenborg et al., 2022). Cronbach’s alpha for the Burnout scale was excellent at both time periods (*ɑ* = 0.88 at time 2 and *ɑ* = 0.90 at time 4). Correlations between Burnout at T2 and T4 were large (*N* = 272, *r* = 0.74, 95% CI [0.68, 0.79]).

### 2.5 Path analysis

IBM SPSS 28 software was used to conduct the preliminary and descriptive analysis. AMOS 28 was used to conduct a path analysis and to estimate the direct and indirect effects. Confidence intervals were estimated by a bootstrapping procedure with 1,000 resampling instances. All confidence intervals are of the percentile type.

### 2.6 Sample size

SEM is a method known to be vulnerable to small sample sizes (Kline, 2011), and the suitable size for conducting analyses is therefore debatable. As a rule of thumb, a sample of 200 or more provides sufficient statistical power for data analysis (Hoe, 2008). Hence, the present sample size of *N* = 272 ought to be sufficient.

### 2.7 Goodness of fit indices

There is a recommendation to use several goodness-of-fit indices (GOF) to assess model fit in Structural Equation Modeling (Kline, 2011). The following GOF indices: The Chi-squared (*χ*^2^) test, Comparative Fit Index (CFI), root mean square error of approximation (RMSEA), Tucker-Lewis Index (TLI), standardized root mean square residual (SRMR), and Akaike’s Information Criterion (AIC) where the best of two competing models would be the model with the lowest AIC value (Akaike, 1974).

A low, non-significant *χ*^2^-value indicates that the covariance matrix of the statistical model is similar to the covariance matrix of the empirical data. A known limitation of this test is the fact that *χ*^2^ is extremely sensitive to large samples of more than 200 respondents (Hoe, 2008). The *χ*^2^ is therefore reported but not used to determine if the model is retained. CFI, TLI, RMSEA, and SRMR have been developed to complement the *χ*^2^-value. Both TLI and CFI are incremental fit indices that compare the proposed model’s fit to the nested baseline or null model. TLI is thought to be resilient against variations in sample size and is highly recommended (Hoe, 2008). A value of 1 indicates a perfect fit, while values of 0.90 to 0.95 or higher indicate an acceptable fit for both TLI and CFI (Hoe, 2008; Kline, 2011). For both RMSEA and SRMR, values closer to 0 indicate a better fit. RMSEA values of 0.05 or lower indicate a very good fit. Values between 0.05 and 0.08 are thought to be acceptable, while values of 0.08 or above indicate a bad fit. An SRMR value of 0.08 or lower indicates a very good fit for the data (Hu and Bentler, 1999).

## 3 Results

### 3.1 Descriptive statistics

Skewness, kurtosis, descriptive statistics, and correlations between the variables can be seen in Table 1. Means, standard deviations, skewness, kurtosis, and correlations for all variables are shown in Table 1.

### 3.2 Path analysis

**Figure 3:**
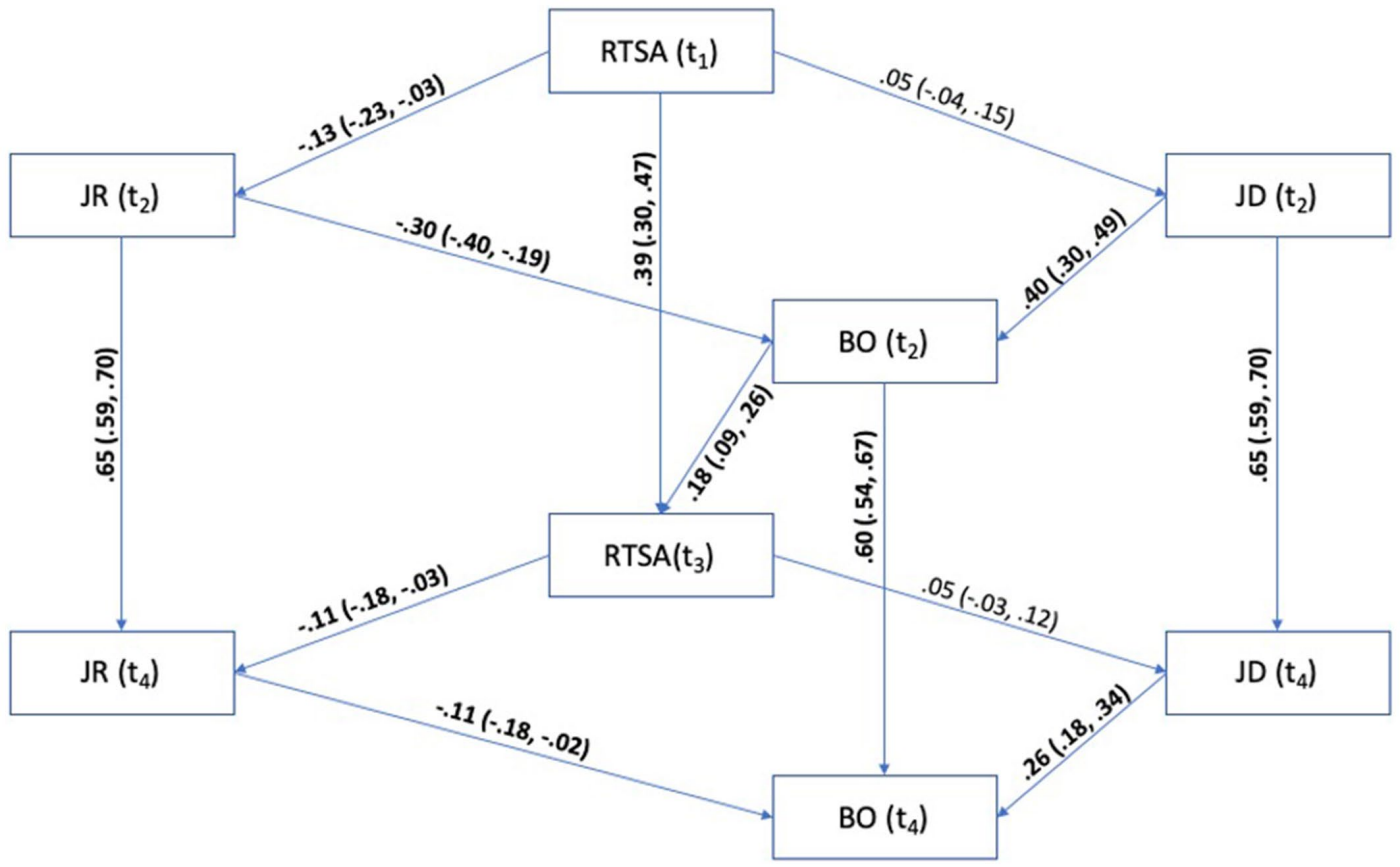
Initial model with standardized path coefficients and 95% confidence intervals. All 95 percent confidence intervals are shown in parentheses. Path coefficients are significant at (*p* < 0.05), marked in bold text. RTSA, Root Transformed Sickness Absence; JR, Job Resources; JD, Job Demands; BO, Burnout.

The theoretical model described in Figure 3, containing all thought paths that represented the hypotheses in this thesis, was constructed and analyzed within Maximum Likelihood Estimation AMOS 28.

The initial model also had a poor fit to the data according to the model fit indices (see Table 2). Modification indices showed that fit could be improved by adding a path between burnout at T2 and job demands at T4. This path also has an empirical justification, as burnout has been found to increase the perceived job demands (Demerouti et al., 2004). We realized that this path should probably be specified in advance, but we simply overlooked this when we designed our model. The added path was statistically significant (*ρ* = 0.12, 95% CI [0.03, 0.20]), and the model fit was improved for Chi, SRMR, and AIC, but not for TLI, CFI, and RMSEA, and the model fit was still not satisfactory (see Table 2).

**Table 2:** Model changes with model fit statistics.

Model	Model change	Chi	df	SRMR	TLI	CFI	RMSEA	AIC
Initial	-	148.2	15	0.163	0.713	0.846	0.181	190.2
2	Add BO T2- > JD T4	142.4	14	0.156	0.704	0.852	0.184	186.4
3	Add *r* between the error terms of JD18 and JR18	62.9	13	0.054	0.876	0.943	0.119	108.9
Final	Add *r* between the error terms of JD19 and JR19	20.3^⊗^	12	0.031	0.978	0.990	0.050	68.2

^⊗^Final model *p* = 0.062, all other models have statistically significant Chi-square values (*p* < 0.05). RTSA, Root Transformed Sickness Absence; Chi, Chi-square test; df, Degrees of freedom; SRMR, Square Root Mean Square Residual; TLI, Tucker-Lewis Index; CFI, Comparative Fit Index; RMSEA, Root Mean Square Error of Approximation; AIC, Akaike’s Information Criterion; r, correlation; BO, Burnout; JD, Job Demands; JR, Job Resources.

Since model 2 (see Table 2) did not adequately fit the data, we checked the modification indices, which indicated that fit would be improved by adding a regression path from job resources at T2 toward job demands at T2 and between job resources at T4 toward job demands at T4. We modeled these relationships by allowing the error terms of job demands and job resources to correlate on both T2 and T4. The reasoning behind this choice was based on three points. First, the JD-R model states that job resources and job demands should be negatively correlated (Bakker and Demerouti, 2007). *Second*, because both job demands and resources were measured at the same time point, it would be difficult to understand the relationship as a causal, non-spurious path. Finally, because the questionnaire at each time point measured the perceived job resources and job demands in the same companies, we surmised that we could expect that the error terms within each time point (T2 and T4) would be correlated as the sample was not randomly drawn from a population but rather represented employees working in the same companies.

**Figure 4:**
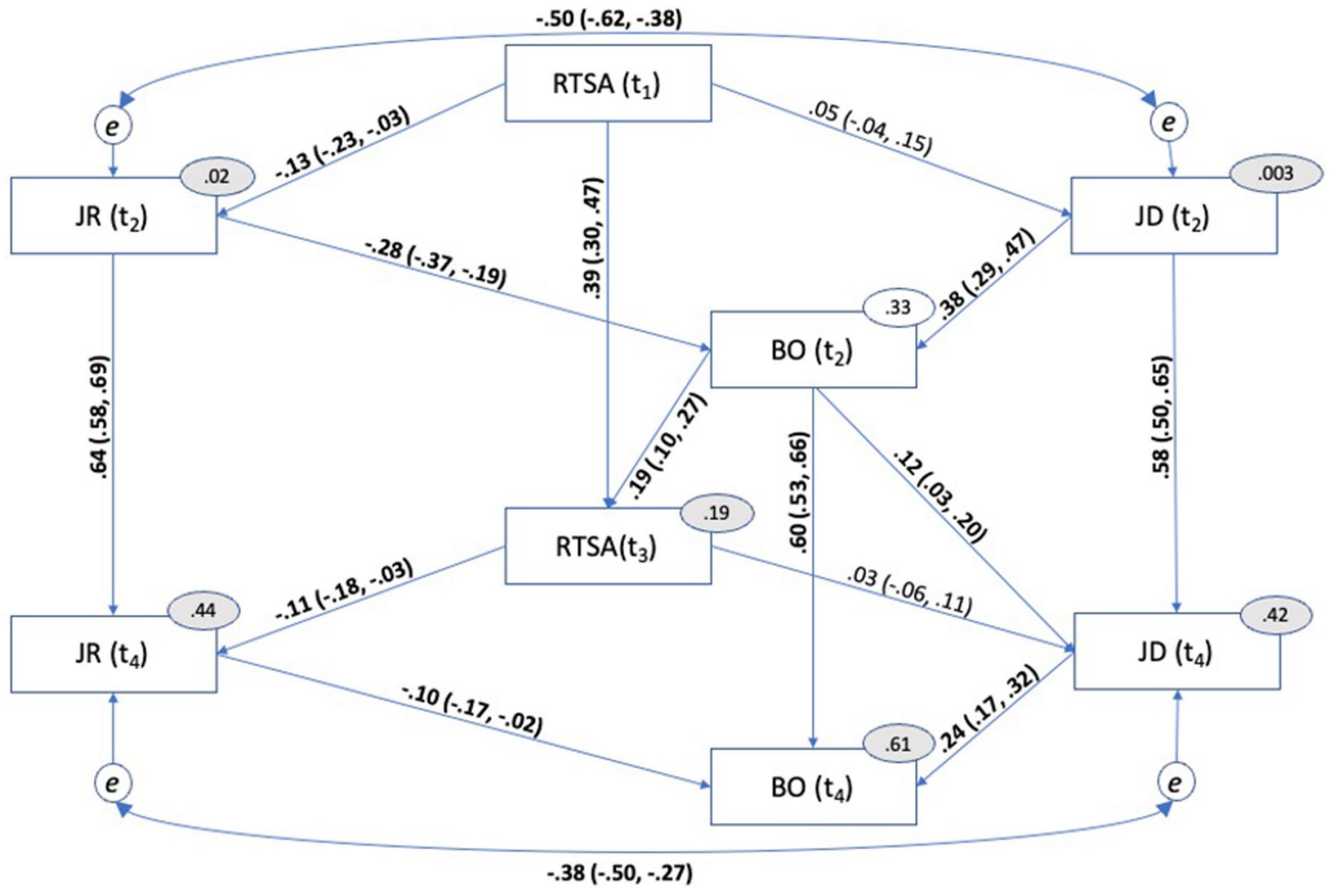
Final model with standardized path coefficients and 95% confidence intervals. All 95% confidence intervals are estimated using the percentile approach in AMOS 28 with 1,000 bootstrap procedures. Explained variance is indicated by grey ovals. CI, Confidence Interval; RTSA, Root Transformed Sickness Absence; JR, Job Resources; JD, Job Demands; BO, Burnout.

The error terms of job demands and job resources at T2 were added first, and then T4. The results of the model fit indices of both these additions can be seen in Table 2, and the final model was a good fit for the data. The final model with path coefficients with a 95% CI is presented in Figure 4.

### 3.3 Assessment of the hypotheses

The indirect effect of job resources on sickness absenteeism through burnout was small yet statistically significant (*ρ* = −0.052, SE*_ρ_* = 0.019, 95% *CI* [−0.086, −0.023]), as was the indirect effect of job demands on sickness absenteeism through burnout (*ρ* = 0.070, *SE_ρ_* = 0.022, 95% *CI* [0.035, 0.105]). Confidence intervals were of the percentile type and were estimated using 1,000 bootstrap samples in AMOS 28. These findings support our hypotheses H1a and H1b, which assume that JD-R theory can be used to predict sickness absenteeism.

Regarding our hypotheses H2a and H2b—that sickness absenteeism will have a positive regression coefficient with job demands—we see in Figure 4 that the regression coefficient is in the expected direction. However, the effect is small and not statistically significant (*ρ* = 0.05, 95% CI [−0.04, 0.15]), and the effect is even smaller when we control for job demands at T2 (*ρ* = 0.03, 95% CI [−0.06, 0.11]). These results indicate that sickness absenteeism only slightly increases the perceived job resources, but the effect is so small that a larger sample will be required for clear identification. In the discussion, we will discuss possible avenues for future research to address these issues.

On the other hand, as we can see in Figure 4, sickness absenteeism reduces the perceived job resources to a statistically significant degree – in accordance with our hypothesis H3a (*ρ* = −0.13, 95% CI [−0.23, −0.03]). This effect remained even after we controlled for the perceived job resources on T2 as expected by H3b (*ρ* = −0.11, 95% CI [−0.18, −0.03]).

This suggests a minor reciprocal effect between sickness absenteeism and job resources, where absence leads to a perceived loss of resources due to reduced workplace engagement.

Regarding the last set of our hypotheses, whether sickness absenteeism at T1 has an indirect effect on sickness absenteeism at T3 mediated through job resources and burnout at T2 (H4a) or mediated through job demands and burnout at T2 (H4b). We supported hypothesis H4a that sickness absenteeism has a positive indirect path through job resources T2 → burnout T2. The effect was estimated to be statistically significant but of small size (*ρ* = 0.005, SE*_ρ_* = 0.003, 95% CI [0.000, 0.012]). Hence, H4a is supported by the data; however, the effect size is so small that it would have a negligible practical effect, which is something we will discuss later. For the indirect path described in hypothesis H4b (job demands T2 → burnout T2), we found a near-zero effect size, which was not statistically significant (*ρ* = −0.001, SE*_ρ_* = 0.004, 95% CI [−0.007, 0.006]). The combined indirect effect through both job demands and job resources was statistically significant with a positive path coefficient, but the effect size was still very small (*ρ* = 0.010, SE*_ρ_* = 0.007, 95% CI [0.000, 0.024]) only explaining 0.01% of the variation in sickness absenteeism.

Finally, sickness absence at T1 had a significant total effect (direct + indirect effects) on Sickness absence at T2 (*ρ* = 0.399, *SE_ρ_* = 0.051, 95% CI [0.31, 0.48]), which is in accordance with previous studies that have found that previous sickness absenteeism predicts 4 to 15% of sickness absenteeism the following year (Roelen et al., 2010) or that the number of sickness absenteeism episodes predicts the extent of sickness absenteeism in the subsequent year (Laaksonen et al., 2013; Reis et al., 2011).

## 4 Discussion

The aim of this study was to (1) conduct a longitudinal test of the JD-R model’s ability to predict sickness absenteeism, (2) explore the reciprocal association between sickness absenteeism and the job characteristics of job demands and job resources, and (3) test whether there is a cyclical relationship between job resources, job demands, burnout, and sickness absenteeism.

### 4.1 Testing the JD-R model’s ability to predict sickness absenteeism

Overall, we found that the JD-R model could predict sickness absenteeism longitudinally and that both job resources and job demands have indirect effects on sickness absenteeism through the burnout construct. The findings indicate that job resources buffer the impact of job demands on burnout, even when we control for previous measurements of burnout. This is in accordance with current empirical evidence (Bakker et al., 2005; Bakker et al., 2010; Xanthopoulou et al., 2007) as well as corresponding with current knowledge in the JD-R model (Bakker and Demerouti, 2007, 2017).

We also found evidence for a statistically significant longitudinal medium-size reciprocal path from burnout in T2 to job demands in T4 after controlling for job demands at T2 (*ρ* = 0.12, 95% CI [0.04, −20]). This finding is in line with other longitudinal evidence showing that burnout affects job demands (Demerouti et al., 2004) and indicates that burnout reduces employees’ ability to cope with them, thereby increasing the perceived level of these job demands (Hobfoll, 2001).

We found that burnout predicted sickness absence with a medium-sized positive path coefficient of *ρ* = 0.19 even after controlling for previous sickness absence. Thus, burnout has a unique predictive effect on sickness absence. The observed correlation between burnout and sickness absenteeism (*r* = 0.21, see Table 1) is comparable to other research on the relationship between burnout and sickness absenteeism, which has found correlations in the range of *r* = 0.21 (Toppinen-Tanner et al., 2005) to *r* = 0.31 (Schaufeli et al., 2009). Thus, our findings are in accordance with previous studies that have shown burnout to be an important antecedent of sickness absence (Ahola et al., 2008; Bakker et al., 2003; Borritz et al., 2010; Hallsten et al., 2011; Toppinen-Tanner et al., 2005; Väänänen et al., 2003). Our findings also expand the scope of the JD-R model, as our data are comparable to those of previous research on the JD-R model and sickness absence, even though we used a different operationalization of the burnout construct. Unlike the standard approach, which typically employs the Oldenburg Burnout Inventory (Demerouti and Bakker, 2008), we used the burnout scale from the COPSOQ II (Pejtersen et al., 2010), translated into Norwegian (Gottenborg et al., 2022).

### 4.2 Reciprocal cycles between sickness absenteeism, job demands, job resources, and burnout

#### 4.2.1 Sickness absence and job resources

As expected by hypotheses H2a and H2b, there was a medium-sized negative path coefficient between sickness absence T1 and job resources T2 (*ρ* = −0.13), and after controlling for job resources at T2, we found a slightly smaller path coefficient (*ρ* = −0.11) between sickness at T3 and job resources at T4. Our results indicate that sickness absenteeism generally leads to a reduction in perceived job resources, which, in turn, contributes to increased burnout (*ρ* = 0.028, 95% CI [0.003, 0.057]). The relationship between job resources and burnout is well understood (Bakker and Demerouti, 2017; Lesener et al., 2019), so we need to provide an explanation for the longitudinal effect of sickness absenteeism on job resources.

Sociological research has discussed whether individuals who are absent from work are being excluded from the social environment at work and in their social network, a hypothesis that has met some resistance (see Bryngelson, 2009). Another vein in sociological research has shown that being unemployed is associated with poorer mental health, as unemployment not only brings worse socio-economic status but also deprives individuals of social status, a sense of collective purpose, and social relations at work (Jahoda et al., 1997). Thus, having a job and being at work involves a number of health-promoting aspects, such as the experience of belonging to a social group, establishing interpersonal relationships, and giving a sense of purpose (Jahoda, 1982; Paul and Batinic, 2010; Rosso et al., 2010). These aspects are parts of the basic social-psychological need for relatedness (Ryan and Deci, 2017). Other aspects of work, such as the experience of mastery in your job, bring about self-efficacy (Rosso et al., 2010) and may be related to the satisfaction of the basic psychological need for competence, like the ability to bring about intended effects at work, which also may lead to a sense of self-determination or autonomy (Rosso et al., 2010) which is important for motivation and wellbeing (Ryan and Deci, 2017).

The job resources measured in this study were *support from colleagues*, *feedback*, *autonomy*, *involvement*, *investment in employee development*, and *leadership quality*. All these job resources would be difficult to obtain when employees are not physically present at work. Hence, being away would entail a lower perception of the available job resources, as we have observed in this study. Thus, in this respect, we would expect that unintentionally being away from work due to illness may reduce a person’s experience of the health-promoting aspects of work.

#### 4.2.2 Sickness absences and job demands

The expected positive path coefficients in H1a and H1b between sickness absence and job demands were found to be in the expected direction on both T1-T2 (*ρ* = 0.05) and on T3-T4 (*ρ* = 0.03). However, these effects were quite small and not statistically significant. Similarly, model fit indices indicated that these effects were not important aspects of a possible causal model for the relation between sickness absence and burnout, and the effects were removed from the final statistical model. One possible explanation for the lack of statistically significant relations between sickness absence and job demands may be the occupational groups in our sample. For people working in manual or physical occupations like day-care centers, personal trainers, chauffeurs, or shop clerks, job demands tend to be non-present when people are not at work. However, for knowledge workers like academics, advisors, or analysts, job demands are mainly of a cognitive nature (Cropley and Zijlstra, 2011), and empirical evidence also indicates that it can be more challenging for these workers to disengage from work than for those employed in manual or physical occupations (Pravettoni et al., 2007). This would be present, especially for high-tenured individuals in high-competence occupations, where few people are ready to take over the work tasks. Thus, for the high-competence workers, job demands (i.e., job tasks) would remain or even grow during a sickness absence. Our sample had many people with jobs where job demands were of a non-cognitive nature. Thus, we could expect that job demands were less present when people were not at work for at least parts of our sample. Unfortunately, we did not collect exact information on the occupation of our sample, so we cannot ascertain whether this fact affected our results.

#### 4.2.3 Reciprocal cycles from sickness absenteeism to future sickness absenteeism

We found evidence for a statistically significant reciprocal cycle in the predicted direction between sickness absenteeism at T1, job resources, burnout at T2, and sickness absenteeism at T3. However, the effect size was tiny (*ρ* = 0.005, 95% CI [0.000, 0.012]), and this effect alone would be of little practical significance as it only explains 0.0025% of the variation in sickness absenteeism at T3. The reciprocal cycle between sickness absenteeism at T1, job demands, and burnout at T2 and sickness absenteeism at T3 was near zero, and the effect was not significant. Finding a large indirect path coefficient becomes more difficult with an increasing number of mediating nodes as path coefficients are multiplied to estimate the indirect path coefficient (Wright, 1934).

### 4.3 Theoretical implications

This study contributes to psychological research by adding knowledge of the longitudinal reciprocal effects of sickness absence on job characteristics, as well as showing that sickness absenteeism may affect future sickness absenteeism through an indirect path through job characteristics and burnout – but that the main path (the one with the highest indirect effect) is through job resources. We believe that these findings point to the importance of including the health-promoting aspects of work in a general psychosocial model of work like the JD-R model. Our study has also expanded the breadth of the JD-R model by using a different operationalization of the burnout construct, thereby showing that the empirical associations of the JD-R model are of a general nature and are not connected to Maslach’s or Demerouti’s conceptualization of the burnout construct (Demerouti and Bakker, 2008; Maslach et al., 1996).

### 4.4 Practical implications

Besides the rather obvious point that leaders and organizations should work to reduce burnout among their employees, our study presents data that show the importance of maintaining employees’ access to job resources even during sickness absence. This is in line with findings on ‘return to work’ (RTW), which investigate factors that affect the time it takes for long-term sickness absentees to come back to work. The general findings show that social relations in the workplace are of particular importance (Tjulin et al., 2010). Experiencing that both leaders and colleagues are supportive and constructive in the meeting with the worker prior to RTW is of particular importance to ensure a swift return to work (Selander et al., 2015; Tjulin, 2010; Tjulin et al., 2010). A recent systematic review found that multiple factors affected RTW, such as social support, attitudes, self-efficacy, and demographic factors (Etuknwa et al., 2019). Supervisors not engaging in their employees’ returning-to-work process might result in a feeling of less support, involvement, and feedback, entailing a feeling of less available job resources, and poor relationships between supervisors and employees returning to work is associated with productivity loss of about 25 percent (Lötters et al., 2005).

Another aspect that might contribute to the observed effect here is the lack of supervisors/leaders to engage in the employee’s return-to-work process and to stay engaged afterward as well. As we found, sickness absence predicted loss of resources, which in turn led to increased burnout. Burnout may, therefore, increase future burnout not only by increasing future job demands but also by reducing the buffer effect of future job resources through sickness absence. When conducting employee surveys, it may, therefore, be beneficial to identify groups of employees who display high levels of burnout and work toward decreasing their job demands and promoting job resources (Gottenborg et al., 2022).

### 4.5 Limitations

This study has some theoretical and methodological limitations that should be addressed. While the original data file included approximately 1,000 respondents, only 272 participants had complete data across four time points. The sample size limits the possible complexity of the model. As a result, we chose to create composite scores for job demands, job resources, and burnout variables. Thus, we could not test the effects of individual job demands and job resources, which is a task for future research.

The majority of the sample were women (70%). Thus, the findings might not generalize to other populations with different gender distributions or across cultures. Whether our findings will generalize is an empirical question. However, we have previously found that the questionnaire used has shown measurement invariance across genders and age groups (Gottenborg et al., 2022). Thus, at least in a Nordic context, we believe that our findings are also relevant to other workplaces.

Another limitation is the fact that we did not have data on occupation, which could be of great interest in seeing the relationship between job demands, job resources, and sickness absenteeism, especially as recent research shows that different professions experience different levels of job resources and job demands (Buvik et al., 2023). Moreover, we lacked data on socio-economic status or other possible moderating factors. Hopefully, this could be handled in future research.

We have used the Norwegian translation of the COPSOQ II Burnout Scale (Gottenborg et al., 2022; Pejtersen et al., 2010). This scale represents a different and more narrow conceptualization of the burnout construct than compared to the Maslach Burnout Inventory (MBI; Maslach et al., 1996) or the Oldenburg Burnout Inventory (OLBI; Demerouti and Bakker, 2008). The COPSOQ II scale has more communalities with the exhaustion facet of OLBI and the emotional exhaustion facet of MBI. We cannot know the extent to which this has affected our results, but one possible redeeming factor is that our correlations between sickness absenteeism and our burnout construct are of similar size as other research that has investigated the relationship between burnout and sickness absenteeism (Toppinen-Tanner et al., 2005).

Another challenge is common to most social science research. We have not modeled a complete causal model, and there exist unmeasured third variables that could work as mediators (e.g., Work Engagement or Job Satisfaction) or as moderators (e.g., occupation or educational background). Therefore, our estimates of total effects and combined indirect effects probably underreport effect sizes for total effects (but not for direct effects).

The study was conducted in Norway, a country with one of the highest sickness absenteeism rates in the world (Proba, 2014). In Norway, employees have smaller economic losses compared to other countries as they are guaranteed full salary from day 1 of the sickness absenteeism and up to a full year. Employers must cover the employee’s salary for the first 16 days, and after that, the Norwegian government covers the salary for a maximum of 1 year. This system creates a safety net for employees who are injured in accidents or experience illness. The public discussions on the reasons for the high sickness absenteeism rate in Norway are ongoing, but no single explanation exists (Fantoft, 2018). Given that the Norwegian social security and sick pay systems are quite unique, it would be interesting to see if the findings from this study can be replicated in countries with different systems. However, despite the Norwegian context and the high national sickness absenteeism rate, we ought to ask whether the structure of our data is based on results from other countries and other contexts. Our results are similar to other results from international samples, and the relationship between burnout and sickness absenteeism that we find is similar to research outside the Norwegian context (Toppinen-Tanner et al., 2005). However, we acknowledge the unique context of the Norwegian sickness absenteeism system, and we believe that it would be of interest to do replication studies in other countries to see the extent to which national or systemic differences contribute to the associations observed in this study.

Some of the scales had Cronbach’s Alpha values of 0.90 or more. While Kline (2011) considers values of *ɑ* ≥ 0.90 as excellent, other researchers have debated whether a high alpha value indicates that one is measuring a narrow aspect of the underlying construct, thus possibly impacting the construct validity if specific parts of the construct are not measured. Some researchers claim that alpha values of 0.95 or higher can be problematic (Hair et al., 2017). The scale “Feedback” had the highest Cronbach’s Alpha in the present study with a value of 0.94. However, removing one or more of its items would result in a scale with less than three items, which is generally not recommended (Raubenheimer, 2004). Thus, we chose to retain all items in our scales.

Another measurement limitation is the right-sided skewness of sickness absenteeism data, which creates challenges for using linear correlation measures. We opted to use the square root transformation (Bartlett, 1936) due to the presence of zeroes in the sickness absenteeism data. According to our analyses, this linearized the associations between the variables to an acceptable extent, but we acknowledge that other methods, such as the use of non-parametric analyses and generalized linear models with Poisson distribution, could also be used, and that future studies should investigate the effect of different methods of handling the skewed distribution of sickness absenteeism.

### 4.6 Future research

While several studies have explored different antecedents of sickness absence, not many have investigated the effects of sickness absence on job demands and job resources. Our study only points to the presence of such an effect. The present study did not differentiate between social resources, task resources, personal resources, and so on, and the same goes for the different components of job demands. It is possible to speculate that different types of resources and demands are affected differently by both short- and long-term absence. Future research should, therefore, aim to investigate exactly which types of resources and demands are related to both well-being and sickness absence. Other aspects that would have improved our current study include knowledge of occupations and educational background to test whether these factors could moderate the associations between sickness absenteeism, job demands, and job resources.

Sickness absence only assessed sickness duration and not sickness frequency. Different types of sickness absence can potentially have different antecedents and consequences, and it would be interesting to see if effect sizes and significance levels change when differentiating between the two. Schaufeli et al. (2009) found that work engagement and burnout do, in fact, affect absence frequency and duration differently, and it would be interesting to explore these findings further.

## 5 Conclusion

This study yields support for several of the propositions in JD-R theory. We found that the JD-R model longitudinally predicts sickness absence in a sample of Norwegian employees. Support for the buffer effect of job resources on burnout was also found, with job resources having large and small to medium effects on burnout. Evidence for reciprocal negative path coefficients from sickness absenteeism to perceived job resources implies that those who are on sickness absenteeism experience lower levels of job resources. This implies that organizations and leaders ought to maintain contact with employees who suffer from sickness absence. Hence, they maintain social relations, contact with leaders and are allowed to involve themselves in their workplace in a way that maintains the experience of job resources. This finding is in accordance with results from the Return to Work literature, where social relations between leaders and co-workers are particularly important for a successful return to work (Etuknwa et al., 2019; Selander et al., 2015; Tjulin, 2010; Tjulin et al., 2010).

## Data Availability

The raw data supporting the conclusions of this article will be made available by the authors, without undue reservation.
